# Hypertension: a lymphatic disease?

**DOI:** 10.1042/CS20245149

**Published:** 2025-06-23

**Authors:** Sunitha Konatham, Bethany L. Goodlett, Hannah L. Smith, Brett M. Mitchell

**Affiliations:** 1 Department of Medical Physiology, Texas A&M University College of Medicine, Bryan, Texas 77807, U.S.A.; †† John Sharp Parkway, Medical Research and Education Building II, Room 2412,Texas A&M University College of Medicine, Bryan, Texas, USA

**Keywords:** hypertension, immunity, inflammation, lymphatics

## Abstract

Hypertension affects nearly one in two American adults and is a major risk factor for cardiovascular events and organ damage. Many treatments exist to lower blood pressure in hypertensive patients; however, their degree of effectiveness varies. It is well known that the pathology of hypertension is interwoven with actions of the immune system. Different types of immune cells infiltrate and accumulate in organs under hypertensive conditions and contribute to cytokine release and persistent inflammation. Recently, it has been shown that blood pressure can be lowered by increasing renal-specific lymphangiogenesis, which facilitates the movement of immune cells out of the kidneys. Hypertension is known to be correlated with increased lymphangiogenesis in tissues including the kidneys, heart, gonads, and skin; however, little is known about this relationship. In this brief review article, we explore the connections between hypertension, a well-studied disease, and the lymphatic system, a historically understudied system. We speculate on points of relation and the potential impact of lymphatic-focused hypertension treatments.

## Introduction

Hypertension is a global health crisis that puts 1.28 billion people worldwide at elevated risk of heart attack, stroke, and organ damage. Existing treatments primarily target the renin–angiotensin–aldosterone system (RAAS) and sympathetic nervous system and have limited success in decreasing blood pressure and improvng patient outcomes [1]. New research is needed to identify additional facets by which hypertension could be controlled.

**Figure 1 A:**
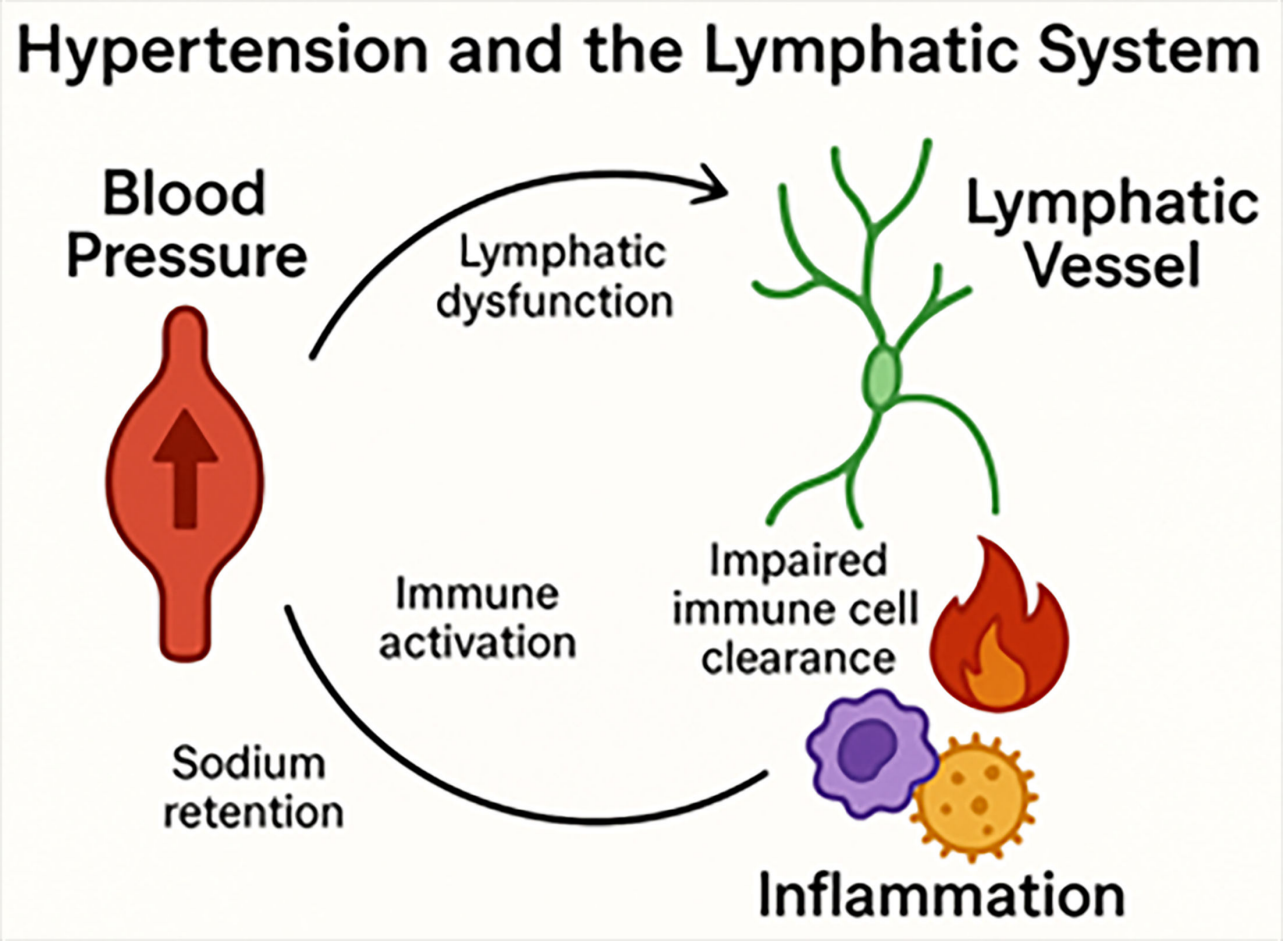
diagram illustrating the relationship between blood pressure, the lymphatic system, and inflammation. These components of the body interact with each other by various mechanisms to disrupt homeostasis and contribute to hypertension.

Hypertension has long been known as a cardiovascular-renal disease; however, recent studies have identified an undeniable connection between hypertension and the lymphatic system (Figure 1). Under hypertensive conditions, increased lymphatic density has been observed in the heart, kidneys, gonads, and skin [2–6]. Additionally, patients with refractory hypertension have significantly elevated vascular endothelial growth factor-C (VEGF-C) serum levels [2]. Although the specific mechanisms linking hypertension and the lymphatic system remain unclear, modulating the lymphatics of hypertensive animals has been demonstrated to affect immune cell populations, inflammation, tissue function, and blood pressure. Augmenting lymphatic density ameliorates hypertension and positively affects the immune environment and organ function in murine hearts and kidneys [7–9]. Furthermore, restricting or impairing lymphangiogenesis exacerbates organ dysfunction and pro-inflammatory immune activities [10].

Lymphatic disorders can impair immune surveillance, rendering individuals more susceptible to infections and possibly contributing to the development of autoimmune diseases such as systemic lupus erythematosus. Lymphatic endothelial cells (LECs) regulate immune tolerance and immune cell trafficking, and their dysfunction may predispose to immune dysregulation. Patients with autoimmune disorders also demonstrate increased prevalence of hypertension, possibly due to shared pathways involving vascular and end organ inflammation and immune activation [11,12]. Hypertensive patients are at higher risk for infections, as well as complications and mortality associated with these conditions [13–15]. Furthering our understanding of the relationship between hypertension and the lymphatic system could uncover new avenues for treatment.

### Current understanding of hypertension & treatment options

Since its discovery, hypertension has been thought to be primarily a cardiovascular problem, with mechanical, autonomic, and hormonal processes all contributing to increased vasoconstriction and/or cardiac output, which eventually leads to elevated blood pressure. Physical narrowing or stiffening of blood vessels due to a variety of factors (e.g. cholesterol deposits in atherosclerosis) requires the heart to pump harder to circulate blood, thus increasing pressure within the arteries [16]. Hyperactivity of the sympathetic nervous system can also contribute to increased blood pressure by causing vasoconstriction and increasing cardiac output [17]. The RAAS is a critical hormonal cascade that regulates blood pressure, fluid, and electrolyte balance [18]. It often becomes dysregulated in hypertension, leading to increased vasoconstriction and fluid retention. Conditions like kidney disease can increase renin release, leading to elevated angiotensin II, which causes vasoconstriction and increased aldosterone secretion. Aldosterone leads to excessive sodium and water retention, raising blood pressure. Because of the cause-and-effect relationships between these processes, they have often been the target for both pharmaceutical and surgical management of hypertension, along with lifestyle factor modifications, such as exercise, diet, and smoking cessation [19].

Atherosclerosis is treated with 3-hydroxy-3-methyl-glutaryl-coenzyme A (HMG-CoA) reductase inhibitors (statins), which lower low-density lipoprotein (LDL) cholesterol levels and stabilize atherosclerotic plaques by interrupting a crucial early step in the cholesterol biosynthesis pathway, reducing the risk of cardiovascular events. Patients with atherosclerosis may also undergo angioplasty or coronary artery bypass grafting to restore myocardial perfusion. Although the Cardiovascular Outcomes in Renal Atherosclerotic Lesions (CORAL) trial did not show broad benefit of renal artery stenting over medical therapy, it remains a viable option in patients with resistant hypertension and confirmed renal artery stenosis [20]. Sympathetic hyperactivity can be attenuated with beta-blockers, which lower blood pressure by blocking the effects of epinephrine (adrenaline) on the beta receptors, slowing heart rate, and decreasing the force of contraction. Alpha-2 agonists stimulate alpha-2 adrenergic receptors in the brain, which reduces sympathetic outflow and lowers blood pressure. Baroreceptor stimulation remains under investigation, with devices like Barostim Neo receiving U.S. Food and Drug Administration (FDA) humanitarian use approval but limited adoption due to mixed clinical trial results [21,22]. In contrast, renal denervation was approved by the FDA in November 2023 for the treatment of resistant hypertension. Trials such as RADIANCE-HTN and SPYRAL HTN-ON MED demonstrated its effectiveness in lowering blood pressure by modulating renal sympathetic nerve activity [23,24].

Hypertension due to RAAS activation can be addressed at each of the steps of the system: renin inhibitors directly inhibit renin and prevent its downstream effects (e.g. aliskiren), angiotensin-converting enzyme inhibitors block the conversion of angiotensin I to II (e.g. lisinopril), angiotensin II receptor blockers block angiotensin II receptors (e.g. losartan), and aldosterone antagonists inhibit aldosterone effects (e.g. spironolactone). Diuretics also help the kidneys eliminate excess salt and water, lowering blood volume and pressure.

### Current understanding of the lymphatic system

Despite the extensive understanding of the different mechanisms that contribute to hypertension and the litany of treatments available for this widespread disease, there are still aspects of hypertension that are yet to be completely understood. These underexplored avenues could be potential opportunities for new therapeutics for hypertension, which would be particularly beneficial for patients who have not responded well to existing treatments. One of these avenues is the lymphatic system - a fluid-based network in the human body whose functions are not yet completely understood, as it is closely integrated with many other bodily systems. It is a network of organs and vessels that works alongside the other organ systems in the body to help maintain homeostasis through functions such as fluid balance, waste removal, and immune function.

The lymphatic system, which is anatomically aligned with the circulatory system, maintains fluid balance by collecting excess fluid that has leaked from the capillaries into the surrounding tissue, filtering it, and returning it to the bloodstream. This prevents swelling (edema) and helps regulate the volume of interstitial fluid in tissues. As lymph nodes filter lymph fluid, they remove waste products, cellular debris, and toxins from tissues to be excreted. Impaired lymphatic function, therefore, leads to fluid accumulation and edema in tissues, as found in a study by Boardman et al. [25]

A study by Randolph et al. explored the role of lymphatic vessels in immune surveillance and found that lymphatic vessels help the body fight off infections by facilitating the trafficking of dendritic cells and lymphocytes to lymph nodes, where they mount an immune response against foreign antigens [26]. When LECs are directly exposed to incoming lymph that is carrying pathogens or antigen-presenting cells that have engulfed pathogens, they activate adaptive immunity by influencing immune cell trafficking, increasing T cell tolerance, and maintaining T cell homeostasis [27,28]. Furthermore, active immune cells seem to control LEC growth and function during inflammation.

Specialized lymphatic vessels in different parts of the body, such as the small intestine and brain, also play active roles in the unique function of these organs, which, in turn, have an impact on the body as a whole. Lacteals, specialized lymphatic vessels in the small intestine, play an important role in lipid transport [29,30]. Lacteals transport chylomicrons, which contain dietary fats, from the small intestine to the bloodstream by absorbing dietary fats and fat-soluble vitamins from the intestine, packaging them into chylomicrons, transporting them through lymphatic vessels, and eventually releasing them into the bloodstream for distribution to tissues throughout the body [29,30].

The lymphatic network in the brain, known as the glymphatic system, serves as an interface between the brain and the body and facilitates the clearance of waste products and excess fluid from the brain’s interstitial space, contributing to brain health and function [31,32]. While this system has many similarities to the peripheral lymphatic system that operates throughout the body, a distinguishing feature is its link to the sleep state and circadian rhythm, with increased clearance during both sleep and the inactive phase [33,34].

### Links between hypertension & the lymphatic system

Pathological lymphangiogenesis is widely believed to be the body’s attempt at ameliorating persistent inflammation by enabling LEC-mediated pro-inflammatory cytokine scavenging and providing additional exit routes to accumulating immune cells; however, these efforts are insufficient to resolve the disease or prevent aforementioned side effects [35]. As with most chronic inflammatory diseases, hypertension is associated with lymphangiogenesis in several different tissue types, including the heart, skin, kidneys, and gonads [2–5]. Furthermore, hypertension is associated with impaired lymphatic function, leaving hypertensive individuals at higher risk for developing lymphedema [36–38].

Together, recent research suggests a role for lymphatic vessels in blood pressure regulation, likely resulting from their contribution to immune cell trafficking and tissue fluid balance. Modulating renal lymphatic density has been reported to decrease blood pressure in mice both before and after the initiation of hypertension [4,8]. Although these findings support an association between hypertension and the lymphatic system, the current body of evidence—primarily derived from animal models—is insufficient to establish a definitive causal relationship. Additional mechanistic studies, particularly in human populations, are necessary to determine whether lymphatic dysfunction serves as a cause or consequence of hypertension.

Hypertension leads to pro-inflammatory immune cell accumulation and inflammation in tissues such as the skin, heart, and kidneys [39,40]. Pro-inflammatory immune cells secrete molecules such as cytokines, chemokines, leukotrienes, and prostaglandins to initiate, maintain, and progress inflammation until resolution occurs. As hypertension is associated with a chronic inflammatory environment, resolution never occurs, and pro-inflammatory immune cells and inflammatory mediators accumulate in tissues, resulting in elevated blood pressure, tissue fibrosis, endothelial dysfunction, oxidative stress, and a positive feedback loop that perpetuates and exacerbates these symptoms. Manipulating certain immune cell populations can prevent and/or ameliorate hypertension but provides a challenge in terms of larger scale immunosuppression and clinical feasibility [12]. Given the increasing prevalence and mortality rate of hypertension, and particularly resistant hypertension, alternative therapies are needed. Modulating the lymphatic system, which plays a critical role in immune surveillance and immune cell trafficking, could provide a way to manipulate the immune system while minimizing these challenges. In this regard, therapeutic lymphangiogenesis presents itself as a promising possibility.

Similar to hypertension, lymphatic disorders are associated with decreases in immune system integrity and increases in pro-inflammatory molecules in tissues and circulation [41]. Lymphatic dysfunction can coincide with losses in nutrients, metabolic deficits, fluid retention, tissue fibrosis, and activation of the innate and adaptive immune systems. Immune cell populations that have been implicated in the pathogenesis of hypertension, including Th1 cells, dendritic cells, and macrophages, have also been implicated in the pathogenesis of lymphedema [12,41]. Given the lymphatic system’s large role in maintaining immunity, it’s not surprising that patients with lymphatic dysfunction are more prone to illness.

Recent studies have demonstrated that granulocyte-macrophage colony-stimulating factor (GM-CSF) promotes hypertension through the activation and recruitment of pro-inflammatory monocytes and dendritic cells [12,42]. These findings suggest that immune modulation can have targeted effects on specific cell populations contributing to hypertensive inflammation. Although adoptive transfer models indicate that myeloid cell trafficking to the kidneys can be reduced through GM-CSF inhibition, additional work is needed to determine whether other immune cell populations are unaffected, which would strengthen the argument for mechanistic specificity. This remains an important area for future investigation to rule out confounding factors such as differential survival or off-target effects and to solidify the causal relationship between lymphatic function, immune cell trafficking, and hypertension.

In addition to immune-related functions, the lymphatic vasculature plays an important role in sodium balance regulation. Sodium is a key regulator of fluid homeostasis and, consequentially, blood pressure. Under hypertensive conditions, sodium accumulates in the interstitium of tissues, where it activates immune cells and serves as a pro-lymphangiogenic signal. Lymphatic vessels transport excess sodium out of tissues and return it to the blood circulation for eventual filtration and excretion. Under homeostasis, this mechanism is typically sufficient to prevent tissue dysfunction and edema. However, under chronic inflammatory conditions, the sodium load may become too high for lymphatics to resolve. Sodium accumulation is associated with increased lymphatic density in the skin under hypertensive conditions [43]. Additionally, mice with augmented renal lymphatic networks show improvements in sodium handling [44].

### Conclusions & future directions

The lymphatic system is complex and intricately linked with other systems in the body, particularly the cardiovascular and immune systems. Based on currently known physiological relationships, there are many targets for future novel therapeutics to alleviate or even prevent hypertension in at-risk populations. The enhancement of lymphatic pumping efficiency and contractility could help improve fluid regulation, potentially reducing hypertension [45]. VEGF-C therapy to promote the growth and repair of lymphatic vessels could improve lymphatic drainage and reduce blood pressure [2]. Anti-inflammatory drugs or biologics can target specific cytokines involved in lymphatic dysfunction, or sodium transport inhibition in the lymphatic vessels could help reduce interstitial fluid retention, and lower blood pressure [46,47].

While not currently commonplace or established as standard practice for hypertension management, further exploration of the relationship between the lymphatic system and hypertension would reveal numerous ways in which lymphatic assessment can be integrated into hypertension management to personalize treatment for a hypertensive patient according to their lymphatic function. Regular monitoring of lymphatic function in hypertensive patients can help in the early detection of any deterioration in lymphatic health, allowing for timely intervention and potentially preventing the progression of hypertension [48]. This can be done through advanced imaging techniques, such as lymphoscintigraphy, near-infrared fluorescence imaging, and magnetic resonance lymphangiography, which can be used to assess lymphatic vessel integrity and function in hypertensive patients [49]. Additionally, identifying and utilizing biomarkers specific to lymphatic dysfunction, such as soluble VEGF receptor-3 and lymphatic vessel endothelial hyaluronan receptor-1, can help in early detection and monitoring of hypertension related to lymphatic abnormalities [50].

Understanding the lymphatic system’s role in hypertension requires comprehensive knowledge of its interaction with these systems. However, there are many gaps in this knowledge, as conducting lymphatic system research has historically proven to be difficult due to a lack of noninvasive tools to measure lymphatic function directly. Most current methods are either invasive or indirect, making it difficult to study the lymphatic system in living organisms, particularly in humans [45]. Most of the current knowledge about lymphatic function and hypertension comes from animal studies. Translating these findings to larger animal systems or humans would be beneficial in terms of accurately portraying clinical implications related to hypertension and lymphatics but is challenging due to species-specific differences in the lymphatic system and its interaction with other systems [48].

Many variables impact the lymphatic system and its ability to be explored through scientific research, including the heterogeneous structure and function of lymphatic vessels, as well as the factors that can further influence this variability, including genetics, lifestyle, and comorbid conditions. Isolating these confounding variables to determine causal relationships between the lymphatic system and other factors is complex, and especially considering that time can also be a variable in chronic conditions such as hypertension, exploring the longitudinal nature of these factors can be difficult as well [46]. However, such longitudinal studies are needed to understand the chronic impacts of altered lymphatic function on blood pressure regulation over time, as well as establishing causal relationships between lymphatic dysfunction and hypertension to help identify whether lymphatic issues precede hypertension or are a consequence of it [2].

By addressing these challenges through advanced research methodologies and technological innovations, the scientific community can enhance the understanding of the lymphatic system’s role in hypertension, potentially leading to novel therapeutic approaches and further integration of lymphatic assessment in hypertension management.

**Clinical Perspectives**

- To connect the interplay between elevated blood pressure, end-organ inflammation, and dysfunctional lymphangiogenesis.
- Evidence suggests that there is a strong interaction between lymphatics in various organs and hypertension.
- Targeting lymphatics in various organs may be a novel therapeutic strategy to combat hypertension in patients.

## Abbreviations

CORAL: Cardiovascular Outcomes in Renal Atherosclerotic Lesions
FDA: Food and Drug Administration
GM-CSF: Granulocyte-Macrophage Colony-Stimulating Factor
HMG-CoA: 3-hydroxy-3-methyl-glutaryl-coenzyme A
LDL: Low-Density Lipoprotein
LECs: lymphatic endothelial cells
RAAS: renin–angiotensin–aldosterone system
VEGF-C: Vascular endothelial growth factor-C
